# Supported self-management in long-term conditions in an African context

**DOI:** 10.4102/sajp.v80i1.1978

**Published:** 2024-04-30

**Authors:** Leigh Hale, Amanda Wilkinson, Sonti Pilusa, Aimee Stewart

**Affiliations:** 1 Department of Physiotherapy, Faculty of Health Sciences, University of Otago, Dunedin, New Zealand; 2 Department of Physiotherapy, Faculty of Health Sciences, University of the Witwatersrand, Johannesburg, South Africa

**Keywords:** Africa, disability, healthcare providers, long-term health conditions, patients, perspectives, state of the art review, supported self-management

## Abstract

Self-management is an important strategy to improve quality of life, appropriately manage long-term health conditions, and reduce the economic burden of long-term health conditions. However, equitable healthcare access remains an issue, and the focus on ‘self’ in self-management is problematic. Our review aims to explore the conceptualisation and evolution of supported self-management in an African context and its relevance to physiotherapy. A state-of-the-art review of the literature was undertaken by the authors. The authors knowledge of the subject area and a database search retrieved recent articles exploring patients’ and healthcare providers’ understanding of supported self-management in Africa. Relevant articles were read, and data summaries of included studies were extracted and tabulated. Findings were organised deductively. Sixteen studies, 11 primary research, and 5 reviews (2016–2023) undertaken in a variety of sub-Saharan countries with healthcare workers (~*n* = 177) and people (~*n* = 16 115) living with a mix of non-communicable and communicable conditions were considered in this state-of-the-art review. Self-management perceptions were drawn from Western authors spanning development research and understanding of the concepts in Western thinking. We conclude that imported concepts, such as supported self-management for long-term conditions, should be considered within local health delivery solutions. These should be embedded in an understanding of traditional African health systems.

**Clinical implications:**

There is a need to develop locally derived African solutions. Self-management strategies for long-term health conditions should be developed, considering traditional holistic African health systems.

## Introduction

Self-management of long-term health conditions is an important concept within healthcare, resulting in extensive global research (Hale, Oosman & Stewart et al. 2022a; Taylor et al. 2014). Touted as an important strategy to reduce the burden of long-term health conditions, self-management has been demonstrated to improve health-related quality of life (Taylor et al. 2014). This state-of-the-art review interrogates and challenges the concept of self-management within healthcare. The review begins by questioning the different terminologies used and the neoliberal individualism perspective in which health self-management is applied. It then focuses on the conceptualisation of supported self-management in an African context. Finally, the review considers why this topic is of relevance to physiotherapy.

## Challenging the concept of ‘self-management’

Although the idea of caring for oneself and, or family to maintain health or treat illness is evident throughout history, several terms, such as self-care and self-management, have been used in the literature, sometimes interchangeably, lacking consensus around their meaning and use (Jones et al. 2011). This is now complicated by the recent addition of terms like self-management support and supported self-management. ‘Self-management’ in health is defined as ‘an individual’s ability to manage the symptoms, treatment, physical and psychological consequences, and lifestyle changes inherent in living with a chronic condition’ (Barlow et al. 2002:178). This involves individuals accessing resources or attending self-management programmes to learn about their condition and acquire skills to manage their health (Taylor et al. 2014).

Self-management is usually packaged in a programme and can be delivered through one-on-one or group interventions in a primary care setting or, more broadly, through population-wide approaches (Jones 2013; Pearson et al. 2007). These programmes generally have two overarching aims: firstly, reduction in the use of healthcare services (emergency and hospital with linked reduction of cost to the healthcare system), and secondly, improvement of health outcomes for people living with long-term conditions (Pearson et al. 2007). Within such programmes, the aim is to influence and positively change patient behaviour. The programme content can vary and may include patient education, coaching, goal setting, skill development (e.g. self-monitoring, self-determination, and problem-solving [De Silva 2011]), planning, and emotional and problem-solving strategy development (Pearson et al. 2007).

Whilst seemingly an attractive strategy for individuals to learn to manage their long-term health, there is an underlying assumption by healthcare providers and health systems that people have the ‘agency or free will and self-efficacy to make daily decisions that would benefit their health’ whilst ‘overlooking the powerful effect of social context’ and that ‘not everyone is in a socio-economic position to prioritise health and be supported to be in control of their health’ (Francis, Carryer & Wilkinson 2018:2). The global advancement in self-management is linked to the increasing cost of healthcare, the reduction in access to healthcare providers and services (including rehabilitation), the impact of long-term conditions on the health of populations, and the associated costs as well as the improvements in quality of life when patients become involved in their healthcare (Mitra et al. 2017). Improvements in quality of life often depend on improving patients’ abilities to manage their physical and psychological health (Pearson et al. 2007). Thus, self-management has become an important determinant of public policy and public spending (Furler, Harris & Rogers 2011) and is underpinned by a neoliberal discourse of personal or individualistic responsibility. ‘Self-management [*is now*] a “policy relevant” construct, clearly within the remit of the health system and [*therefore seen as*] one of the daily tasks of patients and health providers in their encounters’ and may drive further inequities in healthcare (Furler et al. 2011:3).

We argue that self-management in the context of living with a long-term health condition or disability goes beyond the individual, and impacts physically, mentally, emotionally and economically on individuals and their families, community, and the wider society (Mitra et al. 2017). Self-managing health in this context is complex and can be difficult for most individuals but more so for individuals with cognitive impairments such as intellectual disabilities (Hale et al. 2011), mental health issues (Blixen et al. 2016), or those living with multiple and complex long-term health conditions (Francis et al. 2018). Self-management programmes can facilitate people to learn to manage their long-term health condition, but such programmes are frequently inaccessible for many people, arguably for those needing the most support, and often have high attrition rates (De Silva 2011; Furler et al. 2011; Jones 2013). Reviews have identified multiple and complex reasons for inaccessibility. These include geographical, disability, cultural, ethnicity, language and communication, cognition, poverty, housing, income, and employment insecurity, multimorbidity, and potentially living in low to middle-income countries (De Silva 2011; Hale et al. 2022a; Hearn et al. 2019). Critiques of such programmes indicate that health self-management presumes high health literacy and social capital (Ould Brahim 2019; Pickett & Wilkinson 2015).

Self-management support has been conceptualised in two different ways: (1) provision of education, techniques and tools that facilitate healthy decision-making and (2) a person-centred approach enabling a collaborative (reciprocal) relationship that supports the person as they develop the skills needed to self-manage and their self-efficacy to use them, thereby developing over time the person’s confidence to manage their health (Bodenheimer, MacGregor & Shafiri 2005; De Longh et al. 2015; De Silva 2011; Jones 2013; Wyatt & Ampadu 2022). Delivering supported self-management in a collaborative, responsive, and flexible manner may enable a more individualised approach to meet the needs of those previously excluded (Jones 2013). Whilst the literature generally refers to the umbrella term of self-management support (De Longh et al. 2015; Jones, Pöstges & Brimicombe 2016; Taylor et al. 2014), based on that used by De Silva (2011), we have adopted a more differentiating approach to terminology. We suggest that providing the tools or interventions to assist someone in self-managing could be considered ‘self-management support’ and that the responsive and flexible individualistic approach that requires a collaborative partnership be termed ‘supported self-management.’ We further contend that ‘supported self-management’ may be a possible path to support all people in learning to manage their long-term health condition (Rhoda, Smith & Joseph 2017). Four principles are suggested to underpin ‘supported self-management’, namely:

(1) affording people dignity, compassion and respect; (2) offering coordinated care, support or treatment; (3) offering personalised care, support or treatment; and (4) supporting patients to recognise and develop their strengths and abilities to enable them to live independent and fulfilling lives. (De Longh et al. 2015:6)

From our narrative so far, we have contended that the healthcare phenomenon of ‘self-management’ could be reconceptualised as ‘supported self-management’ to enable equitable healthcare access to all living with long-term health conditions. But is this enough? Many would contend that the philosophy of focusing on the ‘self’ is problematic in and of itself (Ould Brahim 2019). Wilson, Wilkinson and Tikao (2022) argue that:

[*S*]elf-management concepts are strongly driven by ideas of personal responsibility and an expectation that a person will learn to manage themselves. This is a cultural artefact, a way of thinking that comes from white, Western, neoliberal countries. (p. 15)

Self-management of long-term health conditions is ideologically desirable from the perspective of neoliberal political and moral economies, which emphasise individuals’ responsibilities regarding their well-being and healthcare (Ould Brahim 2019; Pickett & Wilkinson 2015). Yet the concept of ‘self’ varies across cultures. In cultures that emphasise individualism, people can be considered autonomous (Realo 2003) and thus arguably able to or ‘expected to’ manage their health. Conversely, with collectivism, where the groups people belong to, such as family and community, are highly valued, the ‘self’ becomes less prominent (Realo 2003) and thereby incongruent with the concept of ‘self-management’ of health. Such debates are evolving in nations where the ideologies of collectivism stand out (Basurrah, Al-Haj Baddar & Di Blasi 2022; Wilson et al. 2022). So, what of the African viewpoint? Have the understandings and concepts of supported self-management evolved to encompass an African perspective, and if so, how?

## Evolvement of self-management in the African continent

We undertook a state-of-the-art review addressing why knowledge about supported self-management has evolved in the way it has on the African continent. Underpinning a state-of-the-art review is an understanding that ‘knowledge is shaped by individuals and their community and is a synthesis that will change over time’ (Barry, Merkebu & Varpio 2022:285). Data are collected about a phenomenon and analysed to deconstruct how and why an understanding of the phenomenon has evolved, leading to recommendations for new directions for research (Barry et al. 2022) and clinical practice. A subjective summary presents the argument for ‘where we are now … how we got here … where we could go next’ (Barry et al. 2022). Guided by these questions, a search via Google Scholar was undertaken for any recent article (2016–2023) exploring patients’ and healthcare providers’ understanding of supported self-management in Africa (search terms provided in Table 1-A1, Appendix 1). Authors also retrieved relevant articles based on their knowledge of the subject area. Articles were read for relevance, and relevant data were tabulated about the author, year, setting, population, long-term health condition, aim, method, results, recommendations or clinical relevance and definition of self-management, self-management support or supported self-management.

A total of 16 studies, 11 primary research studies, and 5 reviews (2016–2023) are included in our review, details of which are provided in Table 2-A1, Appendix 1. Studies were primarily undertaken in Malawi, Ethiopia, Kenya, South Africa, Uganda and other sub-Saharan African countries with healthcare workers (~*n* = 177) and people living with long-term health conditions (~*n* = 16 115). Long-term health conditions included Type 2 diabetes (T2D), musculoskeletal pain, chronic low back pain, hypertension, a mix of other non-communicable conditions (e.g. asthma, epilepsy, stroke, and cancer) and communicable diseases (e.g. HIV and tuberculosis). Definitions for self-management, self-management support or supported self-management in these articles were drawn from Western authors (see De Silva 2011), spanning development, research and understanding of the concepts in Western thinking (1999–2014). From the included literature, self-management support currently appears to be driven mainly by a lack of availability of healthcare workers and resources and healthcare accessibility issues. However, healthcare practitioners must acknowledge that patients come with the ability to make their own healthcare decisions. Therefore, this must always be central to any supported self-care strategies that are developed. The clinical relevance of recommendations from included studies suggests that services for people with long-term health conditions in African countries require change at interpersonal, organisational and structural, as well as conceptual levels, with many authors recommending future locally derived solutions be found. Further in the text, we present summarised data extracted from the included studies under the headings of ‘interpersonal’, ‘organisational and structural’, and ‘conceptual’.

### Interpersonal

The included literature suggested that relationships and communication between people were key for facilitating the provision of supported self-management. Relationship development includes improving communication between patients and healthcare providers and deepening the understanding and acknowledgement of peoples’ situations and wider contexts by healthcare providers. Being patient- or person-centred is identified as facilitating supported self-management (Chala et al. 2022; Diener 2021). However, healthcare providers’ attitudes towards and communication styles with patients impact relationships (Angwenyi et al. 2019; Dube et al. 2017; Masupe et al. 2022) with ‘victim blaming’ noted as being unhelpful with people not being able to ‘self-manage by following a set of instructions. …. [*A person’s self-management*] needs to be based on choice and requires the ability to make informed, healthy decisions’ (Iregbu & Iregbu 2016:3). Understanding of one’s own beliefs (i.e. the healthcare provider) about health are suggested to facilitate interpersonal relationship development, with development of communication skills (listening, reflecting, i.e. therapeutic alliance) highlighted as a priority (Diener 2021).

Positive patient–provider (patient- or person-centred) interactions and communication provide an atmosphere whereby patients can engage with the healthcare provider (Angwenyi et al. 2019). Such positive interactions are also suggested to be built on understanding the patient and their wider situation (Diener 2021; Dube et al. 2017; Gumede et al. 2022; Masupe et al. 2022). An acknowledgement by healthcare providers is needed of peoples’ capability to take care of themselves and their family, despite the lack of resources available to them (insufficient healthcare coverage, lack of consistency of access to medications, food, shelter and income) (Angwenyi et al. 2018, 2019; Dube et al. 2017; Masupe et al. 2022). A lack of explanation by healthcare providers is noted to impact engagement with healthcare providers’ advice (Angwenyi et al. 2019), with a disconnect in understanding the impact of wider contextual factors by ‘facility-based’ healthcare providers (Masupe et al. 2022). This includes patients’ traditional health beliefs and needs to be incorporated into supported self-management programmes to ensure their appropriateness and viability.

### Organisational and structural

The need to develop healthcare providers’ understanding of and competency in supporting patient self-management endeavours is evident in the included studies (Angwenyi et al. 2019; Chala et al. 2022; Diener 2021). As the:

[*B*]urden of disability [*was high*] and health resources are limited, [*physical*] therapists have a collective responsibility to educate patients, communities, funders, and policymakers on safe and effective self-management of musculoskeletal pain in South Africa. (Diener 2021:6)

Yet, knowledge in, for example, diabetes care, is ‘not translating into [*nurses*] self-efficacy and self-management support in practice’ (Landu & Crowley 2023:e1).

Health system and service provision challenges exist (Dube et al. 2017), with differences observed in access to the level of care provided to people with, for example, HIV (in primary care) versus participants with non-communicable diseases (NCD) (Angwenyi et al. 2019). Self-management of T2D is affected by poverty, different cultural and religious beliefs, family dynamics and reduced knowledge about self-management practices (Angwenyi et al. 2019). Most studies focus on the medical management of T2D with a compliance focus. None focus on partnership or collaboration between the patient and healthcare provider or explore experiences of people living with T2D daily (Iregbu & Iregbu 2016). Decentralising health services to a primary care level for NCD services in Malawi is suggested to encourage healthcare seeking and decrease reliance on traditional medicine (Angwenyi et al. 2018).

However, primary care is often hampered by inadequate community services and staff shortages, so a recommended solution is to have dedicated community-based multidisciplinary healthcare teams with well-trained healthcare providers (Landu & Crowley 2023; Masupe et al. 2022). However, there is a need to enhance self-management support training for and increase the competence of healthcare providers (Chala et al. 2022; Landu & Crowley 2023). One innovative strategy employed is to train community health workers (CHW) in self-management skills for long-term health conditions to enable them to self-manage their health and those they support in the communities. In-depth interviews found that CHWs feel empowered to manage their health, driven by their wish to help others. Having skills to support their health and confidence in making decisions is essential. These healthcare workers are frequently caught in dilemmas between their comprehensive knowledge of their community and respecting the knowledge they are privileged with from the people they support. Examples of this are disclosing abusive relationships or financial hardships. This predicament impacts CHWs’ effectiveness in delivering community education, informal counselling, social support and advocacy (Majee et al. 2020).

In Malawi, people living with long-term health conditions are offered patient education and counselling in clinic settings and HIV programmes, with support from peer experts and support groups. Lay volunteers from community/faith-based organisations also provide various community-based initiatives, including home nursing, health promotion, adherence counselling and psychosocial support. A qualitative study explored the perceptions of rural people living with a wide range of long-term health conditions regarding the outcomes and benefits of such programmes (Angwenyi et al. 2019). Barriers to the uptake of these programmes include their condition-based nature, which thus excludes many conditions, such as lack of information about membership, cost, physical distance from the group, privacy issues and being too busy. Patient and provider interactions are limited by structural barriers of crowded rooms, lack of privacy, high workload, long queues impeding extensive discussions, lack of staff, limited provision of regular group-based sessions, and staff having limited training and knowledge (Angwenyi et al. 2019). Despite these barriers, participants interviewed had developed skills of self-management such as self-regulation and vicarious learning from others, but their self-management ability was limited by low socio-economic conditions and poor access to health resources, resulting in a heavy reliance on family support. Further, there appeared to be an inequity of resources, with those living with HIV able to access more primary health care support than those living with NCD and those with both HIV and NCD. Angwenyi et al. (2019) conclude that peer–patient and support groups should be increased and that integrated services should be delivered at a primary care level for all those living with long-term health conditions.

A Cape Town, South Africa study set out to develop a couples-focused intervention to improve adults’ self-management of T2D (‘Diabetes Together’) based on the premise that an approach involving a partner motivates and supports sustained behaviour change by the person with diabetes. Using published studies and qualitative interviews with couples, six guiding principles were established, which were (1) providing information and advice on diabetes, (2) helping couples talk about diabetes together, (3) setting goals together, (4) sharing fears and coping with hard times, (5) acknowledging how gender plays a role in diabetes care, and (6) remembering that all couples are different and supporting couples to make choices that fit their lives. This approach must now be piloted (Smith et al. 2023).

Masupe et al. (2022) qualitatively explored barriers and potential solutions to the provision of self-management support provided to people living with T2D or hypertension in a peri-urban township in Cape Town, South Africa, interviewing both the healthcare providers and the recipients of healthcare. Two main challenges reported are patient-based, namely ‘poor patient self-control towards lifestyle modification’ and ‘post-diagnosis grief-reactions by patients’ (Masupe et al. 2022:1). However, poor communication and a disconnect between facility-based services and patients and their families are acknowledged as well as inadequate community care services. Patient-driven solutions include having dedicated multidisciplinary diabetes and counselling services, strengthened family support, patient buddies, patient-led community projects and advocacy (Masupe et al. 2022).

Many interventions to enhance self-management of conditions such as T2D have been found efficacious, but successfully implementing them into practice is challenging, often influenced by national societal and policy contexts. Van Olmen et al. (2022) evaluated the implementation of the ‘Self-Management and Reciprocal learning for Type 2 Diabetes’ (SMART2D) in three countries – rural Uganda, semi-urban South Africa and semi-urban Sweden. Implementing this intervention based on the self-determination theory highlights the contextual impact on delivery, with each country focusing on different aspects of delivery (Van Olmen et al. 2022).

### Conceptual

‘Conceptual’ related to views or understandings of the term ‘self-management’ by authors reporting interpretation of the term in an African context. Self-management is not seen as a panacea for all long-term health conditions (Dube et al. 2017), with optimal self-management needing to be viewed in a larger context (i.e. taking into account social and cultural factors) (Gumede et al. 2022). The Western-based self-management model does not describe all self-care activities undertaken by those living in African countries (Stephani, Opoku & Beran 2018). For some countries, the concept of self-management is considered new and complex to translate (e.g. into Amharic), with overlapping concepts with self-care and self-treatment. There are diverse views of self-management support strategies (Chala et al. 2022). Other authors suggest that opportunities exist to develop and implement contextually adapted, structured self-management support training for healthcare providers (Dube et al. 2017). These should focus on the person, family and community’s ability to activate resources, emphasising living daily with the disease or diseases rather than just on medical management (Iregbu & Iregbu 2016). Other terms used in the West, such as ‘patient empowerment’, require reconceptualisation, especially in resource-constrained public health systems (Angwenyi et al. 2019), such as in African countries. It is noted that people with T2D accept poor health as inevitable and are unaware of the significant health improvements that could be made (Smith et al. 2023). The use of the culturally based mindset of Ubuntu or interdependence, openness, and togetherness is suggested as providing a fertile ground for the success of self-management training and CHW programmes (Majee et al. 2020).

## Discussion

The increasing numbers of people worldwide living with long-term health conditions are placing a growing burden not only on patients and their families but also on the broader community and the health system. The concepts of supported self-management, self-management and self-management support are reasonable recommended ways of dealing with this healthcare crisis but have developed from Western understandings of health and philosophies. The findings from our state-of-the-art review suggest that the concept of self-management has evolved in Africa, as in other parts of the non-Western world, in that what was developed within a Western paradigm was then superimposed into the African context. Like elsewhere in the world, this imported approach has met challenges. These challenges include the definitions of self-management and supported self-management being drawn from Western authors and thus Western conceptual thinking, then implemented because of a lack of available healthcare workers and resources or accessible healthcare. These findings unfortunately support Airhihenbuwa and de Wit Webster’s (2004) much earlier assertion that self-management is a:

Western cultural logic … being masqueraded as a universal truth. There is and remains a reliance on intervention strategies developed for Western countries to ‘solve’ health problems in African countries. (p. 6)

Further challenges include concepts such as patient empowerment, which need examination, especially in resource-constrained public health systems. Behaviour change interventions, for example, motivational interviewing, self-determination theory, enhancing competence in making lifestyle choices, and mindfulness, were likewise imported. These interventions were found to be difficult to implement because of local barriers, such as stigma, entitlement mentality, inadequate community services and staff shortages (Angwenyi et al. 2018; Masupe et al. 2022; Smith et al. 2023; Van Olmen et al. 2022).

Our review found no literature about African traditional medicine or how concepts from this could be included in supported self-management strategies. In South Africa, the *Traditional Healthcare Practitioner Act* was implemented in 2007. However, traditional African healers managed patients with health issues long before Western medicine was introduced to the continent. It is possible that indigenous self-management practices and knowledge were taught to people with long-term conditions that have yet to be acknowledged and reported.

Their approach to healthcare is holistic and not only links cultural and religious beliefs but also involves the psychological, spiritual and social aspects of individuals, families and communities (Truter 2007). Thus, whilst not explicitly stated as supported self-management, many patients in Africa come to healthcare providers with this holistic and all-encompassing understanding and expectation of healthcare. This perspective challenges healthcare providers to ensure that any self-management strategies considered are underpinned by this understanding. If not, the desired behaviour changes may be hard to attain and sustain and, more than likely, be unsuccessful (Airhihenbuwa & de Wit Webster 2004).

When considering the need for African solutions to the long-term management of health conditions, healthcare workers could partner with or at least consider what traditional healers offer patients. Other strategies should include measures to improve patients’ quality of life and the development of self-efficacy to ensure patients’ confidence in managing their own health. Many studies conclude the need for African solutions to manage long-term health conditions, for example, co-created solutions or healthcare workers building their skills in interpersonal relationship development and person-centred care via a strengths-based approach. African solutions could draw on traditional African health strategies to help patients manage their health goals and develop self-efficacy, thus mastering their health care strategies. They could also draw on the African concept of Ubuntu. The term Ubuntu is understood in many African societies; however, many definitions of Ubuntu are often contradictory, with no universally accepted definition (Murithi 2009). Yet, two fundamental aspects of (1) the importance of relationships between people and (2) how these relationships are carried out (undertaken, shown, conducted, or effected), are generally accepted as encapsulating the ‘spirit’ of Ubuntu (Nolte & Downing 2019).

State-of-the-art reviews report the current state and knowledge held of the phenomenon being reported, and then make recommendations for new directions. Seemingly in Africa, imported concepts such as supported self-management and self-management could be considered from the limited literature found and reviewed. However, local solutions drawn from those used by traditional health care providers in deciding what goals of health management patients wish to develop and how they and their families need to solve problems to develop appropriate levels of self-efficacy relating to the health condition need to be considered when developing self-care programmes. An understanding of philosophies such as Ubuntu is necessary to enable those living with long-term conditions to live healthily and well. If a concept is foreign and not understandable, buy-in and the desired behaviour changes will be hard to attain and sustain by both patients and healthcare professionals, and will thus be unsuccessful. So, local researchers exploring and identifying local knowledge would be the main recommended direction from this review.

## Physiotherapy and supported self-management

One last objective requires addressing – what relevance does this have for physiotherapists? Physiotherapists are crucial members of the interprofessional team required to support people living with long-term health conditions; they help to build the skills and confidence for people to self-manage their health and well-being, especially their self-efficacy to manage pain and participate long term in physical activity (Diener 2021; Hale et al. 2022b; Jones et al. 2016). Increasing physical activity is an integral component of managing all long-term conditions, whether it is T2D, persistent pain, stroke, mental health impairments, asthma or HIV (Reid et al. 2022). Whilst physiotherapists have evidence-based ‘tools’ that can be used to help people manage pain, engage in physical activity, and enhance ability and participation, physiotherapists should think beyond the short-term and consider how they can empower people to have agency over their health (Hartley 2019). Physiotherapists, therefore, should, in partnership, also support people in building their self-management skills and confidence to use them, empowering them to control their own health journeys (Jones et al. 2016). People with long-term conditions need ongoing support to live life well, especially those living precariously, and not just education with its current pejorative co-existing concepts of adherence and compliance (Bright et al. 2015; Hale et al. 2022b). Any interventions or programmes developed need to be co-designed with those ‘to whom it matters’ so that the result is contextually appropriate and acceptable to those participating and something of relevance and importance in an African context.

## Conclusion

Our state-of-the-art review explored the conceptualisation and evolution of supported self-management in an African context and discussed the relevance of the topic to physiotherapists. From the limited studies found and reviewed, it seems that imported concepts such as supported self-management and self-management could be considered but that local solutions drawn from traditional understandings of health and the philosophy of Ubuntu are necessary to enable those living with long-term conditions to live healthily and well. Foreign concepts are not always directly ‘transferrable’ nor understandable. Therefore, buy-in and the desired behaviour changes will be hard to attain and sustain and, more than likely, unsuccessful if the cultural understanding of the health of patients does not underpin the development of self-management interventions. Physiotherapists are crucial members of the interprofessional team. They can support people living with long-term health conditions to build their skills and confidence to self-manage their health and well-being, especially their self-efficacy to manage pain and participate, long-term, in physical activity. However, the authors of this review would recommend that local researchers explore and identify local knowledge and facilitate the use of co-design in the development of local solutions.

## Appendix 1

**TABLE 1-A1:** Search terms.

Keywords	Keyword examples
Keywords used in various combinations	Africa, African Disability Health Healthcare provider(s) Long-term condition(s) Patient(s) Perspective(s) Self-management supported self-management, self-management support

**TABLE 2-A1:** Summary of reviewed articles by author, year, setting, population, long-term condition, aim, method, results, recommendations or clinical relevance and definition of self-management, self-management support, or supported self-management.

Author, year, setting, population, long-term condition focus	Aim, method	Results	Recommendations or clinical relevance	Definition of Self-management, self-management support or supported self-management
Angwenyi et al. (2018) Phalombe District, South-east Malawi Patients Communicable conditions (HIV, tuberculosis) and non-communicable diseases (NCDs) (hypertension, diabetes, epilepsy, asthma, stroke, cancer)	Presents findings from open-ended questions from the third round of a longitudinal survey (month 6) and from interviews and focus groups exploring patient self-management experiences/strategies and capacity. Survey participants were recruited from villages in the catchment area attending five community/faith-based organisations. Interview participants – purposive sampling for diversity, drawn from the same catchment area as the survey. See the summary below of a larger study that evaluated patient self-management outcomes (health status, self-management behaviour, and self-efficacy) and the benefits of community-based self-management support initiatives.	Total *n* = 174; Median age >40 years (range 20–84). Over 70% female. Survey *n* = 129; 60% with HIV; most common NCD hypertension, epilepsy, and asthma; Interviews *n* = 10 patients (F = 5) and *n* = 4 HIV expert-patients (F = 1); Four mixed gender focus groups (*n* = 31; F= 2 0; 7–10 people in each group) Discussions held in the local Chichewa language (translated into English). Participant health and care experiences. Past and present health experiences and medication inconsistency impacted the way they took care of themselves. Participant adjustments and self-management practices. Demonstrated capability to manage their conditions despite the resource-constrained setting. Participant developed mechanisms to cope with individualised adaptiveness and linked to coping with circumstances arising in the environment. Heavy reliance on family support and access, or lack of, to resources. Participant experience is shaped by economic hardship and insufficient healthcare coverage. Difference in access for level of care provided to participants with HIV (in primary care) versus participants with NCDs (beyond primary care and public provision). Discouraged healthcare seeking and increased reliance on traditional medicine. Participants with NCDs and HIV comorbidities have multiple barriers to access for knowledge, skills, care, and support.	Decentralisation of integrated health services to a primary care level for non-communicable disease services in Malawi.	*Supported self-management – primarily supported by trained lay people or family:* Day to day tasks that a person undertakes for monitoring, managing condition related symptoms, treatment, and living a healthy lifestyle (Bodenheimer et al. 2002; Richard, Shea 2011).
Angwenyi et al. (2019) Phalombe District, South-east Malawi Patients and healthcare providers Communicable conditions (HIV, tuberculosis) and non-communicable diseases (NCDs) (hypertension, diabetes, epilepsy, asthma, stroke, cancer) Faith and community-based organisations	Explore how the provision of self-management support could contribute to patient empowerment. Q1. What are care providers’ perspectives of chronic disease self-management, and how do they facilitate patient empowerment? Q2. What forms of self-management support exist in community and clinic settings for patients? Q3. Are there differences in patient group self-management outcomes, and if so, why? A concurrent descriptive mixed method study with repeated surveys with patients (baseline, 3, 6, and 12 months) was conducted to evaluate self-management outcomes, and qualitative interviews/focus groups were conducted with patients and healthcare providers. The survey was adapted from a Chronic Disease Self-management programme, behavioural risk factor questions from a Malawian study, and modified questions from other literature. Included Likert and semi-structured questions. Pretested with *n* = 20 patients from the same area.	Patients total *n* = 185 Survey *n* = 140 baseline; F = 102; Mean age 42 years (range 20–84): interviewed patients T2 *n* = 128; T3 *n* = 129; T4 *n* = 126. Interviews *n* = 14; F = 6; Mean age 42 years (range 35–70); HIV experts mean age 43 years (range 33–47). Focus groups *n* = 31; F = 20; Mean age 54 years (29–73). Healthcare providers (representation by gender, responsibilities of clinical, non-clinical, and health managers), total *n* = 13; F = 6; Mean age 42 years (range 26–55). Healthcare provider perspective of self-management – patient ability to manage conditions on their own with active support from family and other community caregivers; role was to impart knowledge about symptom identification, medication, and clinic appointment adherence, and behaviour change, and increase understanding that disease(s) are incurable; Access to healthy food, shelter, and income impacted on ability to provide guidance and on the patient ability to self-manage; Patient/provider interactions limited by structural barriers of crowded rooms, lack of privacy, high workload, long queues impeded extension of discussion; healthcare provider attitude and communication style; lack of staff limited provision of regular group-based sessions; training and knowledge limited; unprepared nonclinical staff; inability to follow up patients. Positive patient provider interactions provided an atmosphere where disclosure could occur by patients. Patient engagement with advice was hampered by a lack of explanation of what sort of exercise they could do, and those with physical impairments wanted to know what exercises they could still engage in, BSL not screened regularly, seasonality, and cost of fruit and vegetables. Community self-management support provided by community-based organisations, peer to peer support (based on HIV support group meetings). Barriers were absence of groups for other conditions, lack of information about membership, cost, physical distance from group, privacy issues, too busy. Diversify information channels and restructure patient-support groups however mixed response to all-inclusive groups. Separate groups minimise distortion of health messages, difference in treatment regimens requiring separate discussions. Wanted groups to provide access to financial support and developmental assistance.	Changes required at interpersonal, organisational, and structural levels to facilitate patient empowerment. Patient level factors, care providers competence, and exposure to and receipt of self-management support resources will be determinants of realising patient empowerment in rural African settings. Stark contrast is evidenced between well-resourced disease-specific interventions (HIV) and general health services. Reconceptualisation of patient empowerment in resource-constrained public health systems is required.	*Supported self-management – primarily supported by trained lay people or family*: Self-management is a patient’s ability to manage conditions independently with support from family, friends, and other community caregivers. Patient empowerment in chronic disease management requires broadening perspectives and critical examination of the role of healthcare professionals and other community-based providers (Bodenheimer et al. 2002; Richard, Shea 2011).
Chala et al. (2022) Ethiopia Healthcare providers (doctors & physiotherapists) from three hospitals Chronic low back pain (CLBP)	Explore how healthcare providers in Ethiopia understand and conceptualise self-management and provide self-management support for people with CLBP. Interpretive descriptive qualitative approach. Undertaken and analysed in Amharic. Translated into English at Braun & Clarke’s fourth step to engage the rest of the team in analysis.	Total Healthcare providers *n* = 24; median age 24 years (24–42 range); M = 17; Physiotherapists *n* = 12. Self-management was a new concept, complex to translate into Amharic, with overlapping concepts with self-care and self-treatment. There are barriers to the provision of self-management support, and there are diverse views of what self-management support strategies entail. Self-management has patient and health system benefits. Patient-centeredness is key to facilitating self-management support. Specific competencies are required to facilitate self-management support.	Lots of opportunities to design self-management support strategies for people with CLBP in Ethiopia. Need to enhance self-management support training for and increase the competence of healthcare providers.	*Self-management* Self-management support enhances patient engagement in care decisions and skills for managing conditions. Refer to Adams and Markus 2004; Lorig & Holman 2003; Newman, Steed and Mulligan 2004; and Lorig et al. 1999.
Dube et al. (2017) South Africa Health service providers (working in different system levels) and patients. Two public primary healthcare facilities (community health centre with 24 h casualty and maternity service and primary healthcare clinic (weekdays), urban township, Tshwane metropolitan area Chronic conditions (diabetes, hypertension, asthma, epilepsy, and HIV)	Explore the knowledge, attitudes, and self-management needs and practices of patients with chronic diseases to improve community health nurses’ knowledge and skills when working with patients. Qualitative exploratory design with content analysis. Focus groups, interviews, and observations. Interviews/focus groups translated to English and peer review of two transcripts undertaken for control accuracy.	Health service workers total *n* = 12 (clinic *n* = 4; programme managers *n* = 4; academics/experts *n* = 4). Patients total *n* = ~80; Focus group interviews two/facility (*n* = 32; F = 23); Individual interviews (*n* = 2; F = 1); 10 observations individual consultations (*n* = 6; F = 4); and health education group sessions in waiting area (*n* = ~30–40; four groups); in preferred indigenous language. Five issues for patients with chronic diseases – health system and service provision challenges, healthcare provider attitude and behaviour, adherence to medication and lifestyle, patients’ personal and clinic experiences and self-management tool preferences.	Opportunity to develop and implement contextually adapted, structured self-management support interventions for community health nurses in developing countries. Self-management is not a panacea for all chronic diseases.	*Self-management / Self-management support* Actions that individuals and caregivers take for themselves, children, families, and others to stay fit and maintain good physical and mental health, meet their psychological needs, prevent illness or accidents, care for minor ailments and long-term conditions, and maintain health and wellbeing after an acute illness or discharge from hospital (De Silva 2011). Self-management support – educating patients to maintain greater control through understanding their condition, being involved in monitoring and action, complemented with support in goal setting, decision making, adopting a healthy lifestyle, and knowing when to seek help.
Gumede et al. (2022) uMkhanyakude district, rural KwaZulu-Natal, South Africa Grandparents Chronic diseases	Explore chronic disease self-management practices and challenges of grandparent caregivers. Qualitative research design, interpretive paradigm using 18 repeat in-depth interviews.	Grandmother caregivers (*n* = 6); Aged 56–80 years old; caring for 2–15 grandchildren. Used Self-management Framework (focusing on illness needs, activating resources, living with a chronic illness – tasks and skills within each category, Schulman-Green et al. (2012). Living with chronic illness – processing emotions, adjusting to illness and new self, integrating illness into daily life, meaning making. Focusing on illness needs – following instructions, completing health tasks, performing health promotion activities. Activating health resources – healthcare, spiritual, family, and community resources.	Optimal self-management must be viewed in a larger context (social and cultural factors). Self-management practices used by grandmothers are inextricably linked with grandchildren caregiving. Self-perception of caregiving meant grandmothers were responsible for the primary caregiving of grandchildren when they needed care, lived in poverty, and had conditions requiring self-management. The caregiving role brought meaning, but illness management was difficult when relationships were strained. We must include young people in self-management education to support grandparents’ efforts.	*Supported self-management:* Individual’s ability, in conjunction with family, community, and healthcare professionals, to manage symptoms, treatments, lifestyle changes, and psychosocial, cultural, and spiritual consequences of chronic conditions (Richard & Shea 2011).
Landu and Crowley (2023) King Sabata Dialindyebo subdistrict, OR Tambo district, Eastern Cape, South Africa Primary healthcare Nurses Diabetes	Evaluated diabetes knowledge, self-efficacy, and performance of diabetes S-MS. Cross-sectional correlation design survey. Self-administered questionnaire in three parts: i) demographics, ii) Diabetes Basic Knowledge Test (DBKT) (Ledbetter 2011), iii) Self-efficacy and performance in self-management support (SEPSS instrument) (Duprez et al. 2016).	Total *n* = 100; 86% female, 59% with nursing diploma, 10% with Postgraduate Certificate Primary Health Care. Age range 24–61 years. High DM knowledge (mean 11.9 out of 14) and self-efficacy scores (mean 18.91 out of 24). Performance S-MS (mean 17.81 out of 24). Knowledge not associated with self-efficacy or performance. Self-efficacy has a strong positive correlation with the performance of S-MS (r = 0.78, *p* < 0.01). Nurses with PG qualification higher DM knowledge (mean 92.9 vs 83.8, *p* = 0.03). More years of experience weak positive correlation with performance of S-MS (r = 0.21, *p* = 0.05). DM knowledge not translating into self-efficacy and S-MS performance in practice.	Nurses need support to implement self-management support through guidelines, education, training, mentoring and an integrated chronic care system (*see SA ICDM Model, which talks about ‘assisted self-management’*). Discussion – audit patients educated in waiting areas or during consultation and systematic review: self-management support has the most impact in primary care settings, self-management support should be provided throughout the care continuum, integration of self-management support can be classified by engagement of HCP (high active, medium sometimes, low aware self-management support provided elsewhere). Patients who perceived they had a high level of engagement with their healthcare professionals had better health related outcomes.	*Self-management support:* Ongoing process where individuals are actively involved on daily basis in the management of their chronic condition. Draws from work such as Lorig, Wagner Bodenheimer (America), Holman (Australia), Barlow (UK) and others, spanning 1991–2003.
Majee et al. (2020) Genadendal and Greytown, rural South Africa Community health workers (CHW)	To understand the motivation for participating in self-management training, the skills gained, and the perceived impact of training on CHW health behaviour personally and professionally. Participated in *Act Healthy programme* (Schopp et al 2015) and in-depth individual interviews, thematically analysed (Braun & Clarke 2006).	Total *n* = 20 (F = 19). All self-identified as Coloured. Age Mean 40 years old. Employed as CHW Mean 7 years. The male participant completed training and demographics but did not interview. Motivation for participating – desire to learn and help others. Skills gained – goal setting and action planning, self-awareness, and confidence. Perceived health behaviour impact – solution implementation, peer support and new behaviour sustenance.	Culturally based mindset – Ubuntu or interdependence, openness, togetherness -provides fertile ground for success of self-management training and CHW programmes. While effective, in-depth knowledge of the community may be problematic – open book, what happens in the family is known in the community and may affect outreach, community education, informal counselling, social support, and advocacy. Difficult to disclose abusive relationships or financial hardships.	*Supported self-management – primarily supported by trained community health workers:* Self-management is a collaborative effort between individuals, families, and healthcare professionals to manage symptoms, treatments, lifestyle changes, and psychosocial, cultural, and spiritual consequences of health conditions (Richard & Shea 2011). Social Learning Theory, self-efficacy. Three core tasks of self-management are behaviour and emotional management of changes in role brought on by illness (Lorig & Holman 2003).
Masupe et al. (2022) Peri-urban township in Cape Town, South Africa Two sites Healthcare professionals and patients Diabetes and hypertension	Explored experiences, barriers, and solutions for enhancing self-management from patients and healthcare providers. In-depth interviews with healthcare providers and focus groups with patients.	Healthcare professionals total *n* = 8 (health promoter [1], dietician [1], clinical nurse practitioner [4], social worker [1]). Site A – M *n* = 1; F *n* = 4; Site B – F *n* = 3. Four patient focus groups total *n* = 43: 1) *n* = 13, F = 9, M = 4; 2) *n* = 10, F = 10; 3) *n* = 8, F = 6, M = 2; 4) *n* = 12, F = 10, M = 2. Main challenges – lifestyle modification for disease self-management, poor partnerships with HCPs and family, post-diagnosis grief, cultural norms, poor HCP-patient communication, and disconnect between facility-based HCPs and patients’ lived experiences. Barriers to SM – stigma, entitlement mentality, inadequate community services, and staff shortages.	Solutions to enhance self-management: Dedicated community based multidisciplinary healthcare team with well-trained healthcare professionals. Healthcare professionals are free to use patient-centred consultation models (motivational interviewing). Pre and post counselling services in the community for patients and families. Chronic disease buddy system (like HIV one). Chronic disease programmes featuring patient driven community projects and supporting patient led advocacy.	*Self-management …. Self-management support*: Self-management is described based on tasks of medical/behavioural management, role management, and emotional management (Lorig & Holman 2003).
Van Olmen et al. (2022) Uganda, South Africa, Sweden Diabetes Primary healthcare facilities	SMART2D Project – paper reports analysis of the implementation process and interaction with context from a community perspective. Evaluation based on Medical Research Council Guidelines and taxonomy of implementation outcomes. Designed in collaboration with research teams and stakeholders in each country, resulting in a cultural fit for each setting.	Uganda – *n* = 9 primary healthcare facilities in rural communities Iganga and Mayuge Cape Town – *n* = 2 semi-urban community health centres in Khayelitsha Sweden – *n* = 2 socioeconomically disadvantaged urban communities with a high proportion of immigrants in Stockholm. Societal and policy context influenced implementation in all three countries and impacted implementation. Countries focused on in-depth implementation in accordance with feasibility and relevance in the local context. Identified key uncertainties and context specific questions.	Key elements of designing an intervention: aspects of implementation of organisation or structure, absorptive capacity, implementation feasibility and fidelity, capacity, and organisational context of the implementation team and or recipients.	*Self-management support*: Assumptions underlying SMART2D – Self-determination Theory, Community mobilisation, Peer support trained in person centred techniques (motivational interviewing for autonomy, relatedness, and competence in making lifestyle choices).
Smith et al. (2023) Cape Town, South Africa Couples Diabetes	To develop a couples-focused intervention to improve adults SM of T2DM – Diabetes Together. Used a person-based approach. Phase 1 – interviews with people living with DM to find out SM barriers and facilitators, synthesised with information from existing interventions, background research, and theory to develop guiding principles and logic model. Phase 2 – prototyped intervention and interviews with couples to optimise intervention.	Phase 1 – Defined intervention target behaviours to motivate behaviours of treatment adherence, improve diet, and increase physical activity. Two core papers were drawn on related to couples’ perspectives on DM management (Lister et al. narrative review, Gupta et al. review). Twenty interviews *n* = 10 couples, with one partner having diabetes (*n* = 5 couples with one female person with DM; *n* = 4 couples spoke English & Afrikaans; *n* = 6 couples spoke isiXhosa). Median age 51.5 years. Themes identified from interviews – knowledge, fears, external stressors, substance use, moods, food & physical activity, libido. Literature review – three existing interventions for T2DM self-management in SA. Phase 2 – two half-day workshops developed with diabetes status and single gender groups for some topics, adapted some resources from elsewhere. Contains didactic, participatory elements and mindfulness. Videos. Held talk aloud sessions with nine couples (*n* = 5 M with T2DM, *n* = 8 couples primary language isiXhosa, median age 48.5 years) viewing 4–9 intervention sections per session. Refined intervention.	Phase 1 – Developed *six guiding principles* for overcoming intervention context barriers and motivating participants to undertake target behaviours. Also developed logic model to explain how the intervention would work. Phase 2 – developed first couples focused intervention. Suggest approach involving a partner motivates and supports sustained behaviour change by a person with DM. People with DM do not recognise significant health improvements that can be made and accept poor health as inevitable. Now need to pilot for feasibility and acceptability.	*Self-management support – support from spouse*: Not defined – Model of psychological capability, physical and social opportunity, autonomic and reflective motivation. Interdependence theory – lack of awareness of health threats and shared motivation to overcome health issues, poor relationship quality and communication.
**REVIEWS**				
Diener (2021) Adults Musculoskeletal pain	Narrative literature review (methods not discussed) to identify characteristics in the patient for successful engagement in self-management and in the (physio) therapist for supporting patient self-management. To inform evidence-based support programmes for self-management of musculoskeletal pain.	Attributes of therapist – communication skills of listening and reflecting, therapeutic alliance, and motivational interviewing; educational skills of addressing psychosocial barriers to engagement in a self-management programme, self-reflection on own beliefs, health, and pain neuroscience education; physical rehabilitation skills of person-centeredness, needs tailored, and consideration of aspects to improve patient adherence. Attributes of patient – readiness for behaviour change, health locus of control, pain beliefs, and self-efficacy. Assessments – Pain Stages of Change Questionnaire, Health Locus of Control, Pain Catastrophising Scale, Tampa Scale for Kineisophobiae11, Pain Self-efficacy Questionnaire, Patient Health Questionnaire.	Self-management support programmes should prepare participants for behaviour change but also be in place to provide support during the self-management development period. Therapists need to improve their skills in neurophysiology and pain-related psychosocial research. Communication skills training of therapists needs to be a priority. As the burden of disability and health resources are limited, therapists have a collective responsibility to educate patients, the community, funders, and policymakers on safe and effective self-management of musculoskeletal pain in South Africa.	*Self-management support*: Self-management support involves the provision of interventions to identify obstacles to behaviour change and prepare and support people to safely and effectively manage health conditions and maintain quality of life.
Hearn et al. (2019)	A scoping review of self-management of non-communicable diseases in low- and middle-income countries, which included six African countries.	Type 2 Diabetes Egypt (2017) Hypertension South Africa × 2 studies (both in 2016) Type 2 Diabetes Mali (2018) Type 2 Diabetes Nigeria (2017) Stroke Ghana (2018) Heart failure Uganda (2016)	SM appeared to be effective in improving physiologic indicators, patient self-care, and/or patient quality of life in the short term. Most interventions were based on booklets, SMS or phone-based calls, and educational sessions. Authors recommended that future SMS should be co-developed with local patients, communities, and clinicians.	*Self-management support:* Self-management support – educational booklets, SMS messages, peer-led education sessions, education received via a smartphone application.
Iregbu and Iregbu (2016) Africa Adults Diabetes	Explain the concept of self-management, examine African diabetes self-management literature, highlight gaps in knowledge, and present implications for research. Method not stated. Provides a narrative review. Studies reviewed dated 2000–2015.	Self-management of DM in Africa – affected by poverty, different cultural and religious beliefs, family dynamics, and reduced knowledge about self-management practices, results in poor glycaemic control. Most studies focused on medical management of diabetes with a compliance focus. None focused on partnership or collaboration between person and healthcare professional. None explored experiences of people living with diabetes on a day-to-day basis.	Cannot self-manage by following a set of instructions. It must be based on choice and requires making informed, healthy decisions. Victim blaming will not assist. Focus on the ability to activate resources and live daily with the disease rather than just medical management.	*Supported self-management*: A process where individuals are actively involved in their disease management (McCorkle et al. 2011; Richard & Shea 2011).
Otanga et al. (2022) Kenya and Uganda Primary healthcare, community urban, peri-urban, and rural settings Adults Diabetes	Scoping review summarising use and efficacy of peer support and social networking interventions in diabetes self-management. Followed by Levack et al. (2011) and Arksey and O’Malley (2005).	Total *n* = 13 studies (2013–2021); Kenya (*n* = 11); study designs RCTs or pre/post design (*n* = 4), retrospective or cohort (*n* = 4), qualitative (*n* = 3) and one each of mixed-method and cross-sectional. Peer review focus (*n* = 11); role of social networks (*n* = 2). Total *n* = 7317 participants; intervention length (3–12 months); delivered by nurses, peer supporters/educators/leaders, and community health workers, multidisciplinary teams. Intervention outcomes are summarised by learning, behavioural, clinical, and other outcomes. Most showed improved HbA1c, blood pressure, eating behaviours, physical activity, and social support. Intervention efficacy is summarised by assistance in daily management, social and emotional support, links to clinical care and availability of ongoing support. Most interventions are effective.	Intervention efficacy challenges – recruitment (clinician rather than patient-driven), reduced feasibility and acceptability (lack of member input), attrition/retention, places to meet, an ongoing social connection beyond clinic meetings, poverty (explored microfinance using phone networks for virtual support), low literacy, and poor eyesight. Some interventions demonstrated sustainability through the involvement of multiple stakeholders in design and delivery and by working within existing healthcare systems and networks. Reduces cost to and stigma for patients. Creates synergy. Suggests evidence for peer support and social networking for diabetes care (like HIV systems).	*Advocating for Supported self-management:* Not stated. State self-management is a preferred option for meeting the lack of trained healthcare workers and affordable healthcare. State few diabetes support interventions explore means of improving self-management within social support networks.
Stephani et al. (2018) Sub-Saharan African countries Adults Type 2 Diabetes	A systematic review (type not stated) – reports the status of self-management of people with diabetes in Sub-Saharan Africa and presents analysis on the extent of following recommended self-management behaviours as defined by the American Diabetes Association (healthy eating, being active, monitoring, taking medication, reducing risk, psychosocial aspects). Added alternative medicine/healers. Risk of bias and quality was assessed using quality assessment tools for cross-sectional studies (Guyatt, Feeny & Patrick 1993), pre-post studies (National Institutes of Health), and randomised controlled trials (Cochrane).	Total *n* = 43 studies undertaken prior to Sept 2016 in Nigeria (*n* = 13), South Africa (*n* = 11), Ghana (*n* = 6), Uganda (*n* = 4), Ethiopia (*n* = 3), Cameroon (*n* = 2), one each in Tanzania, Kenya, Sudan, and Zimbabwe. Study types – observational (*n* = 35), longitudinal (*n* = 1), and experimental (*n* = 6 with 2 studies describing the same intervention). Total *n* = 8281 participants; F = 4676; Age >50 years old; length of time with DM >5 years Total *n* = studies reporting healthy eating behavioural information (21), physical activity behaviours (17), monitoring blood glucose levels (15), medication management (26), risk reduction (15), psychosocial aspects (3), and alternative medicine/healers (11). Self-management of diabetes in Sub-Saharan Africa is insufficient (lack of physical activity, insufficient risk reduction, medication and nutritional adherence, inability to monitor blood glucose). Western based model of self-management does not describe all self-care activities. Structured diabetes self-management education programmes are effective. Limited exploration of psychosocial aspects.	The review did not consider staff shortages or lack of medicine. Could support structured diabetes self-management programmes with health solutions. Include psychosocial factors into studies/programmes (stress, family support). Consider those who have not been diagnosed but have type 2 diabetes.	*Self-management:* Not defined. Analysis of self-management behaviours as per American Diabetes Association (healthy eating, being active, monitoring, taking medication, reducing risks, psychosocial aspects).
